# Maternal singing and speech have beneficial effects on preterm infant’s general movements at term equivalent age and at 3 months: an RCT

**DOI:** 10.3389/fpsyg.2025.1536646

**Published:** 2025-01-29

**Authors:** Manuela Filippa, Gianluca Filippa, Elisa Della Casa, Alberto Berardi, Odoardo Picciolini, Sara Chiara Meloni, Clara Lunardi, Alessandra Cecchi, Alessandra Sansavini, Luigi Corvaglia, Didier Grandjean, EVC Group, Fabrizio Ferrari

**Affiliations:** ^1^Department of Psychology and Educational Sciences, University of Geneva, Geneva, Switzerland; ^2^Independent Researcher, Aosta, Italy; ^3^Neonatal Intensive Care Unit, Women’s and Children’s Health Department, University Hospital of Modena, Modena, Italy; ^4^Department of Medical and Surgical Sciences of Mother, Children and Adults, University Hospital of Modena, Modena, Italy; ^5^Pediatric Physical Medicine & Rehabilitation Unit, IRCCS Ca’ Granda Ospedale Maggiore Policlinico, Milan, Italy; ^6^Department of Neurosciences, Psychology, Drug Research and Child Health, Careggi University Hospital of Florence, Florence, Italy; ^7^Division of Neonatology, Careggi University Hospital, School of Medicine, University of Florence, Florence, Italy; ^8^Department of Psychology “Renzo Canestrari”, University of Bologna, Bologna, Italy; ^9^Neonatal Intensive Care Unit, IRCCS AOU Bologna, Bologna, Italy; ^10^Department of Medical and Surgical Sciences, University of Bologna, Bologna, Italy

**Keywords:** prematurity, maternal voice, general movements, early intervention, sensorimotor system

## Abstract

**Background:**

General Movements (GMs) are part of the spontaneous movement repertoire and assessing them helps to determine the integrity of the central nervous system in newborns. The aim of this study was to investigate the effects of maternal singing and speaking in the Neonatal Intensive Care Unit (NICU) on preterm infants’ GMs at term equivalent age and at 3 months.

**Methods:**

In this multi-center randomized clinical trial, 56 stable preterm infants (25–32 weeks) were randomized to either an intervention group – in which mothers were asked to speak and sing to their infants for 20 min, 3 times per week, for 2 weeks – or to a control group. GMs were recorded both before and after the intervention – which took place at term equivalent age – and again at 3 months corrected age. The GMs were blindly coded based on the muted video tracks to produce both a general score and a detailed score.

**Results:**

Before the intervention, there were no significant differences between the two groups. The intervention wielded a significant effect on the GMs general score (*p* < 0.05). The effect was also marginally significant for the GMs detailed score (*p* = 0.06). To avoid influencing outcomes, future research should control for differences in maternal engagement outside of the intervention.

**Conclusion:**

Live maternal singing and speaking are fundamental human practices that, in this study, enhanced preterm infants’ general movements and potentially supported their neurobehavioral development. Integrating and supporting early vocal interaction into routine NICU care is crucial for at-risk populations.

## 1 Introduction

Worldwide, rates of preterm birth have barely changed in recent decades, with an increase only in defined areas (Ohuma et al., 2023; Organization, W. H, 2023). In 21 European countries, the preterm birth rates in 2020 were, on average, 4% lower than the rates predicted based on trends from the previous 5 years, with comparable estimations across all socioeconomic groups (Zeitlin et al., 2024).

Despite advancements in neonatal care leading to improved survival rates, prematurity remains associated with various health challenges. These include both short-and long-term complications for the newborns and their families, as well as a broader societal burden (Ashorn et al., 2023; Blencowe et al., 2012).

Among a subset of extremely preterm babies (22–26 weeks’ gestation), reported survival without neurodevelopmental impairment at 2 years ranges from 20 to 42% (Moore et al., 2012; Serenius et al., 2013; Younge et al., 2017). In very and moderately preterm babies, the rates of survival without severe or moderate neuromotor and sensory disabilities at 2 years corrected age, exceed 90% (Lugli et al., 2021; Pierrat et al., 2017). While the incidence of cerebral palsy in preterm babies is decreasing (under 10%), rates of severe functional disability and cerebral palsy are higher in neonates with a lower gestational age (Lugli et al., 2021; Pascal et al., 2018; Pierrat et al., 2017). These preterm children are also at higher risk of developing minor neurological dysfunction: a typical neurological profile includes difficulties with posture, muscle tone, regulation, and balance, as well as mildly abnormal reflexes, coordination, and cranial nerve function; these issues may affect everyday functioning and can also be associated with learning and cognitive problems (Broström et al., 2018). Early parental interventions in the neonatal period, such as skin-to-skin contact (Moore et al., 2012) during the first weeks of life in the NICU, represent a promising approach to improving the later developmental outcomes of preterm infants (Spittle and Treyvaud, 2016), with positive, clinically meaningful effects extending to the age of 36 months (Vanderveen et al., 2009).

Preterm birth, especially with gestational age at or below 32 weeks, increases the risk of language delay (Barre et al., 2011), affecting one in four and one in three very preterm children at 30 months and 42 months corrected age, respectively (Sansavini et al., 2010). Language difficulties have also been identified in less immature 24-month-old preterm children (Charkaluk et al., 2019; Sansavini et al., 2021) and can persist in preterm children up to school age, with cascading effects on literacy and educational attainment (Guarini et al., 2016; Putnick et al., 2017; van Noort-van der Spek et al., 2012).

The causes of atypical developmental trajectories in preterm infants extend beyond medical factors. Environmental influences, such as atypical sensory inputs incompatible with the developing brain’s needs and sensorimotor development, play a significant role. Additionally, repeated painful procedures and early separation from caretakers during NICU admission contribute to an accumulation of stress factors (Cheong et al., 2020; Filippa et al., 2019b).

To reduce neonatal stress, individualized care practices are increasingly being implemented in the NICU. These measures include regulating harmful sensory stimuli, introducing non-pharmacological pain management strategies, and actively involving parents in the care of hospitalized neonates (Beltrán et al., 2022; Hassankhani et al., 2020; Waddington et al., 2021).

The publication of specific guidelines developed by an interdisciplinary group of experts (van den Hoogen, 2019), including pediatricians, parents, neonatologists, psychologists, and therapists has greatly furthered the process of active family inclusion, although the presence of parents on hospital wards still varies greatly from facility to facility (Kokorelias et al., 2019).

### 1.1 Maternal singing and speech in the NICU: the vocal contact

Recent systematic reviews have shown that when hospitalized preterm infants hear their mother’s live voice directed towards them, this improves their physiological stability, has positive effects on their nutritional status, and helps reduce maternal anxiety, thus also benefiting the parents (Bieleninik et al., 2016; Filippa et al., 2017; Provenzi et al., 2018). Early and constant exposure to the voice of a parent in a multimodal context such as skin-to-skin contact can have both short-and long-term effects on infants’ linguistic abilities (Caskey et al., 2014). In the long term, increased exposure to live directed speech during a preterm infant’s stay in the NICU enhances parental word count and positively impacts preterm infant’s linguistic scores at 2 years (McGowan et al., 2024). These gains may be underpinned by early neural mechanisms, such the enhanced neural discrimination of sound changes (Kostilainen et al., 2021) and more marked responses to deviant sounds (Partanen et al., 2022).

Maternal speech and singing during a painful procedure also act as analgesics, reducing the expression of pain and increasing oxytocin levels, significantly so in the case of singing (Filippa et al., 2021b); at the same time, they boost the mothers’ own levels of oxytocin thereby mitigating maternal anxiety (Filippa et al., 2023b).

In terms of longitudinal research, the Cochrane review by Haslbeck et al. (2023) acknowledges the potential of musical and vocal interventions to benefit preterm infants, though it highlights that current evidence regarding their direct impact on developmental outcomes, such as those assessed by the Bayley Scales of Infant and Toddler Development, is of low certainty and inconclusive (Haslbeck et al., 2023). However, a small number of interesting studies have shown that hearing the maternal voice – recorded or live – can have significant effects on the preterm infant over the longer term. In one set of studies for example, the transmission of recorded maternal voices via bone conduction was associated with significantly improved neurofunction in preterm infants (Picciolini et al., 2022) at 3 months corrected age (Picciolini et al., 2014), though it yielded no significant difference at 6 months corrected age. Another study on the long-term effects of early voice exposure showed that the quantity of live adult speech in the NICU was positively correlated with infants’ early social development (face preference) at 7 months corrected age, suggesting potential long-term effects of voice/speech exposure at neonatal intensive care facilities. Moreover, early exposure to voices plays a pivotal role in facilitating brain changes (Romeo et al., 2018) that in turn support cognitive, language, and motor development in early childhood (McGowan et al., 2024). Finally, early exposure or deprivation to voices remains crucial for key processes like auditory processing which is foundational for broader neurodevelopment (Adam-Darque et al., 2020; Filippa et al., 2023a). Notably, when newborns hear voices, as opposed to silence or instrumental music, sensorimotor areas and salience networks—brain regions associated with memory, cognition, and motor function—are activated (Loukas et al., 2024). Taken together, the aforementioned studies suggest that vocal exposure may influence infants’ neurodevelopmental trajectories and may serve as a compensatory mechanism to mitigate neurodevelopmental delays in preterm infants, although its effects on specific developmental domains warrant precise investigation.

### 1.2 General movements assessment

General Movements (GMs) are a type of spontaneous motor activity that is frequently studied in fetuses, preterm and term-born neonates, and young infants (Prechtl et al., 1979). It is now widely accepted that the brain and central nervous system can generate a vast repertoire of endogenous activities even in the absence of specific stimuli. GMs are generalized movements that involve the entire body in a varied sequence of movements. They begin and end gradually. They have been characterized as limb rotations and motor trajectories effected with elegance, continuity, and fluidity (Prechtl, 1990). GM patterns are part of the repertoire of spontaneous motor behaviors, and their origins, characteristics, and clinical function as early markers for neurological deficits have all been extensively described in the scientific literature (Ferrari et al., 1990; Prechtl et al., 1997). Abnormal general movements, in particular, have been identified as reliable markers for the early detection of later neurodevelopmental disorders (Bosanquet et al., 2013). The GMs assessment has been widely used in the preterm infant population as a non-invasive method for evaluating young infants’ neurological development, and indeed preterm infants carrying high neurobiological risk may already exhibit abnormal GMs during the first few days of life (De Vries and Bos, 2010).

In some cases, the quality of preterms’ GMs normalizes over the first weeks of life or before term age equivalent (Cioni et al., 1997; De Vries and Bos, 2010). In other cases, particularly when birth weight is low, preterm infants’ movements continue to differ from those of full-term infants (De Vries and Bos, 2011). Preterm infants exhibit less varied movements than their term-matched counterparts, resulting in a lower complexity index.

“Fidgety movements” are specific, small-amplitude movements of the neck, trunk, and limbs that typically emerge between 6 and 9 weeks after term birth and can be assessed starting at 3 months of age (Einspieler and Prechtl, 2005). These movement patterns manifest as micro-movements of the neck, trunk, and limbs with different rates, directions, and accelerations (Prechtl et al., 1997).

The absence or rarity of fidgety movements between 3 and 5 months is a significant marker of risk for severe neurological deficits (Einspieler et al., 2015).

This randomized controlled trial is part of a larger investigation examining the effects of early vocal contact between parents and preterm newborns in the NICU (Filippa et al., 2021a). Multiple assessment measures were used to evaluate the short-and long-term effects of this early parental intervention on the maturation of the newborns’ autonomic nervous system (Filippa et al., 2024b) and their long-term linguistic development (Filippa et al., 2021a).

The present study evaluates the effects of the vocal contact—through singing and speaking—on the general movements (GMs) of preterm infants during NICU hospitalization, assessed at term equivalent age and 3 months of corrected age. The primary aim was to enhance early vocal contact between parents and preterm infants in the NICU, with the secondary aim of improving neurodevelopmental outcomes as measured by GM assessments. The study tested the hypothesis that a structured maternal vocal intervention would be positively associated with better GM scores at both time points.

## 2 Methods

### 2.1 Study sample

In this randomized controlled study, 56 preterm infants born between 25 and 33 weeks gestational age (GA), were recruited at four sites in Italy between February 2019 and November 2020. The inclusion criteria for the study stipulated that the newborns were required to have been born at the participating center, with a gestational age at birth of between 25 and 33 weeks, a birth weight that was appropriate for their gestational age (>3rd percentile, <97th percentile), a birth cranial circumference greater than the 10th percentile, and an Apgar score of >7 at 10 min. At the time of enrollment, newborns were expected to be in a stable condition. They were either spontaneously breathing or receiving respiratory support via a low-flow nasal cannula. Infants requiring respiratory support with a high-flow nasal cannula, non-invasive ventilation, or invasive ventilation, as well as those experiencing hyperbilirubinemia requiring exchange transfusions during hospitalization, were ineligible for participation.

Infants with congenital malformations and/or genetic abnormalities, as well as those diagnosed with severe conditions such as periventricular leukomalacia grade III and IV, intraventricular hemorrhage grade III and IV, or severe sepsis, were excluded from the study. Additionally infants whose parents did not sign an informed consent form were also excluded. Furthermore, mothers were excluded from the study if they exhibited evident depressive symptoms, had a history of drug abuse during pregnancy, or were under 18 years of age. The psychologists stationed in the participating NICUs conducted assessments for maternal post-partum depression. In the presence of one or more risk factors for depression, standardized protocols were implemented and, where appropriate, the mother was referred to the perinatal psychiatric specialist to explore the need for pharmacological intervention. Mothers who were assessed for mental health issues were not included in the study. Finally, mothers were also excluded if their level of Italian language proficiency precluded them from completing the study questionnaires and from fully understanding the informed consent form. The level of maternal education was assessed and no significant differences were found between the two groups.

### 2.2 Study setting

The participating healthcare facilities were Bologna University Sant’Orsola-Malpighi Hospital (Bologna), NICU Fondazione IRCCS Ca′ Granda Ospedale Maggiore Policlinico, Careggi University Hospital (Florence), and Modena University Hospital (Modena).

All these centers are third-level units providing the same level of care to seriously-ill newborns. The four NICU levels range from Level 1 (the mildest) to Level 4 (the most severe). At the third level, fragile preterm newborns require the support of hi-tech incubators and mechanical ventilators and usually spend long periods in the NICU to ensure stabilization and recovery before hospital discharge. All the centers in the study apply the principles of Family-Centered Care as defined by the European Standards for Newborn Care (Kostenzer et al., 2022). These standards underscore the importance of nurturing the well-being of both the newborn infants and their families. At the core of these standards is a commitment to providing 24-h access to the unit for both mothers and fathers, thereby fostering an environment of inclusivity and support. The standards also prioritize effective pain management strategies for newborns, recognizing the importance of minimizing discomfort and optimizing comfort levels. Finally, they emphasize the importance of catering for the needs of parents by creating a supportive environment for families.

At 6, 12, and 24 months corrected age, the children selected for participation in the study underwent routine general neurological examinations (EON) conducted by trained researchers. The final EON assessment yielded a positive or negative rating. When an infant obtained a final negative score, they were excluded from the current analyses.

The clinical characteristics of the sample are reported in Table 1.

**Table 1:** Clinical characteristics of the intervention and control groups.

Variables	Intervention (*M, SD*)(*n* = 29)	Control (*M, SD*) (*n* = 27)	*p*
Gestational age at birth (weeks), *M (SD)*	29.84 (1.97)	29.98 (1.59)	0.39
Birthweight (grams), *M (SD)*	1201.38 (264.66)	1305.30 (269.05)	0.15
Apgar score at 5 Min, *M (SD)*	7.45 (1.68)	7.25 (1.77)	0.75
Mother’s age (years), *M (SD)*	36.79 (4.22)	34.88 (6.96)	0.23
Postmenstrual age at intervention start (weeks), *M (SD)*	34.05 (1.56)	34.35 (1.27)	0.48
Weight at intervention start (weeks), *M (SD)*	1683.66 (342.43)	1770.19 (298.38)	0.33
Postnatal age at intervention start (days), *M (SD)*	29.28 (18.04)	31.89 (14.90)	0.59

### 2.3 Study design

Centralized patient randomization was conducted using a secure, web-based randomization system, via which the research investigators at each center registered new patients and obtained intervention arm assignment. Stata statistical software was used to build a block allocation sequence with various block sizes stratified by GA at birth and gender (StataCorp, College Station, TX, USA). In the case of twins, randomization was applied at the family level such that sets of twins (8%) were assigned to the same group.

The study was independently approved by each center’s Ethical Committee: Modena Health Authority’s Independent Ethics Committee of Area Vasta Emilia Nord (P.0006292/18); Bologna Health Authority’s Independent Ethics Committee (P.348/2018/Sper/AOUBo); Milan Mangiagalli CE (P.205); and Florence Careggi Hospital CE (P.77/2017). The infants’ postnatal data was recorded for further analysis. The study was conducted in accordance with the ethical standards of the Declaration of Helsinki (Association, W. M, 2013) and written parental consent was obtained for all participating infants.

### 2.4 Power analysis

This study is part of a larger research project designed to investigate the effects of early maternal vocal interventions on various neurodevelopmental and physiological outcomes in preterm infants. The sample size for the present study was calculated based on the primary outcome measure: heart rate variability (Filippa et al., 2024b). To account for an estimated 20% attrition rate, the target sample size was set at 80 participants (40 per group). This sample size is in concordance with the specific literature, which supports similar population sizes for assessing the effects of early interventions on preterm infants’ general movements (Spittle et al., 2015).

### 2.5 The live vocal intervention

The intervention group mothers were asked to talk (10 min) and sing (10 min) to their infants three times a week for 2 weeks, over the hour following afternoon feeding. The mothers were asked to speak in their own language and then to sing familiar songs while observing their baby’s reactions. They were asked not to touch their infant during the intervention itself. The order of the two vocalizations, speaking and singing, was strictly alternated across the six sessions.

The intervention began once the inclusion criteria had been met. For further details, see Postnatal Days at Intervention Start and Postmenstrual Age at Intervention Start in Table 1. Additional information such as the median values and percentiles 25–75 are included in Supplementary Table S1.

The intervention took place on the hospital ward, while the infants were in active sleep, quiet wakefulness, or active wakefulness phases, but not in deep sleep or crying. This meant that deep sleep and sleep cycles were not disrupted by the intervention. Also, in stable preterm newborns, quiet wakefulness is known to be the most favorable condition for family members to initiate early sensory interventions and engagement (Westrup, 2007).

We encouraged the mothers to position their faces in front of the incubator opening, as close to their infant’s head as possible. Concurrent medical procedures were not permitted during the intervention to optimize adherence to the protocol.

The mothers in the intervention group were guided by a formal protocol devised by the first author of the manuscript and by the research supervisor, who is the last author. The instructions were clear and straightforward. The mothers were directed to engage in both talking and singing, but in two distinct phases of the intervention, preferably using their mother tongue (although they were free to choose their own preferred language). They were allowed to freely select the content of their conversation and songs.

While the mothers were encouraged to discuss topics of their own choice, where suggestions were sought, the researcher recommended conversations about their home routines or future family plans post-hospitalization. Regarding the singing, the researchers were allowed to make suggestions, upon request, such as choosing familiar and emotionally resonant songs sung during pregnancy or songs embedded in the mothers’ musical memories. Researchers also conveyed that mothers could opt for wordless humming, and that repeating the same song multiple times was acceptable, all while attentively observing the babies’ behaviors and reactions.

The researchers at each of the centers ensured that the protocol was followed correctly by being present during the recording of the sessions. If the mothers preferred privacy, the researchers viewed the recordings afterwards in order to discard any sessions where mothers failed to communicate vocally. No sessions were excluded from the final sample.

For the first time in research on early vocal contact, we also collected data from an active control group. The mothers assigned to this group were encouraged to spend the same amount of time by the incubator as the intervention group mothers, while observing their infant’s behaviors based on a standard set of cues. This control condition was designed both to include the silent live presence of mothers next to the incubator as a benchmark for comparison as well as to actively include these mothers in the study.

The active control group mothers engaged in an observational program, focusing on observing their babies’ behaviors. They were instructed to refrain from speaking or singing only during the observation sessions (20 min per day, three times per week, for 2 weeks). Outside of the observation sessions, standard parental and medical care continued as usual.

The intervention and control sessions were all video recorded, and adherence to the protocol was verified for both groups by a trained researcher.

### 2.6 Video recording of spontaneous movements

Digital video recordings of the infants’ movements were made before the intervention, between 32-and 34-weeks GA, at the end of the intervention, at term equivalent age, and again at 3 months corrected age. Of the planned 168 recordings (56 × 3), 14 are missing due to technical or logistical difficulties. Each video recording lasted 3–5 min. The following protocol was put in place to ensure consistency of measurement. The infant was placed in a supine position in a semi-open nest, wearing a nappy and a baby bodysuit. It was recommended to lay the infant on a blue cloth for the purposes of filming. All infants were in a waking state; given that behavioral states are not always well defined at low gestational ages, the researcher waited until the infant initiated movement to begin recording. The use of a pacifier was not allowed during the recording of the infants’ general movements (GMs) because this could have altered the quality of the GMs.

The camera was placed on a tripod about 1.5 m high, about 50 cm above the infant’s incubator. All centers used a camera of the same make and model.

### 2.7 GMs assessment

General movements are gross movements involving the entire body and are identifiable from the early fetal stage up to 3–5 months post-term. According to Prechtl’s method of assessment (Einspieler and Prechtl, 2005), two distinct patterns of normal general movements may be observed: writhing and fidgety movements. Writhing GMs occur preterm, at term, and up to 6–8 weeks post-term, and are of small to moderate amplitude and slow to moderate speed. Fidgety movements, which may be observed from 6 to 20 weeks post-term, are small-amplitude, moderate-speed, and multi-directional movements of the neck, trunk, and limbs, and are continual in the awake infant. Abnormal patterns of general movements include so-called poor repertoire general movements (PR), which occur between term age and 6–9 weeks and are a monotonous sequence of movements that lack the complexity that distinguishes normal GMs. A second type of abnormal GMs are labelled cramped-synchronized GMs (CS): these movements appear rigid as opposed to smooth and fluent, and the infant’s limbs and trunk contract and relax almost simultaneously (Ferrari et al., 2002; Ferrari et al., 1990).

For the purposes of the present analysis, three or four episodes of GMs (excluding periods of crying) were selected and first digitalized and then edited using Adobe Premium Pro software (v.2.0 for Windows). At each of the participating centers, a blinded researcher made a compilation of the GMs video recordings at each of the three ages and stored each compilation in a separate file.

The quality of the GMs was independently analyzed for each infant by two observers, an experienced GMs trainer (FF, see authors) and an experienced GMs researcher (DE, see authors). The coders were blind to the infants’ pre-, peri-and postnatal medical history, clinical status, and group assignment. DE was totally blind to the imaging, outcomes, and perinatal history of the infants from the centers in Pisa, Milano and Bologna. Interobserver agreement was 92%. To resolve any disagreement in coding, the GMs of the individual case were jointly reviewed, and consensus was reached after discussion.

The assessment of GMs relies on visual Gestalt perception.

Gestalt perception is a sophisticated visual technique used to assess complex phenomena (Lorenz, 1971). In the general movement assessment, it allows to capture their overall qualities, including complexity, fluency, and elegance. This approach relies on the observer’s ability to perceive patterns holistically rather than focusing on individual details, making it an irreplaceable tool for evaluating the quality of movements in preterm infants (Prechtl, 1990). Among trained and experienced coders, inter-rater reliability is strong, ranging from 89 to 93% (Einspieler and Prechtl, 2005). In this study, the blinded assessment of the individual video compilations yielded two types of outcome: a Global score and a Detailed score. The global score was based on the assessment of writhing and fidgety movements according to the standard methodological principles of Prechtl’s approach to evaluating the quality of GMs (Einspieler and Prechtl, 2005). The global analysis thus involved the recognition and coding of normal (writhing or fidgety) versus non-normal GMs (i.e., poor repertoire, cramped-synchronized, or absent or exaggerated fidgety GMs). More specifically, three distinct categories were assessed both before the intervention (T0) and after the intervention (T1): cramped-synchronized GMs (CS), poor repertoire GMs (PR), and Normal GMs (N)(for details see Table 2). At 3 months corrected age the global score corresponds to the assessment of fidgety movements via three categories, namely Normal, Abnormal and Absent Fidgety Movements (for details see Table 3).

**Table 2:** GMs global scores at T0, before the intervention, and at T1 after the intervention.

Group	Time point	CS	PR	*N*	NA	Total
Intervention	T0	0	6	22	1	28
Intervention	T1	0	8	18	3	26
Control	T0	2	7	16	2	25
Control	T1	1	8	16	2	25

CS, cramped-synchronized GMs; PR, poor repertoire GMs; N, normal GMs; NA, not available.

**Table 3:** GMs global score (Fidgety Assessment) at 3 months corrected age.

Group	Time Point	Normal FMs	Abnormal FMs	Absent FMs	NA	Total
Intervention	3 months	24	0	0	5	29
Control	3 months	18	1	1	7	27

FMs, Fidgety movements; NA, not available.

The detailed scoring for the writhing period (which spans both preterm and term age) is based on the assessment of the individual components of general movement (Einspieler and Prechtl, 2005). A refined procedure for scoring these components has been proposed by Einspieler et al. (2015). The detailed score reflects the quality of the various components of GMs, such as amplitude, speed, special range, proximal and distal rotations, onset and offset, and tremulous and cramped components of the upper and lower extremities. The optimality concept may be applied to the detailed evaluation of GMs to yield an optimality score (GMOS). The GMOS quantifies the detailed characteristics of GMs (Einspieler et al., 2016), with a score of 42 indicating maximum performance both at T0 before the intervention and at T1, after the intervention.

At 3 months corrected age, the detailed evaluation of GMs encompasses the assessment of five additional subcategories to yield the Motor Optimality Score (MOS), ranging from a maximum of 28 points (optimum performance) to a minimum of 5 points (worst possible performance).

The detailed assessment and corresponding optimality score help to identify early markers of cerebral palsy during the neonatal period (Einspieler et al., 2019).

### 2.8 Environmental measures

Background noise levels at each of the participating NICUs were monitored using a calibrated sound level meter (Voltcraft Phonometer SL-10; Conrad Electronic, Hirschau, Germany). Three sets of measurements were taken: two before the intervention began, one inside the intervention room and one inside the incubator, and one during each session, at 10 cm from the infants’ heads. This last measurement was required to guarantee that the infant could hear the mother’s voice (which needed to be at least 10 dBA louder than the background noise). These values were not subjected to any further analysis.

### 2.9 Statistical analysis

Differences between groups (intervention vs. control) and over time (i.e., T0 vs. T1 and T2) were tested using nonlinear mixed effect models estimated with the lme4 R software package (Bates et al., 2014). Nonlinear mixed effect models are particularly suitable for small sample size and when normality assumptions are violated. Moreover, while we acknowledge that small sample size remains an issue of this study we partially overcame this problem by adopting a permutation approach (i.e., permutation t-test to assess differences among clinical characteristics between intervention and control groups at T0; see Table 1).

Intervention and time were viewed as fixed effects, while the subjects were treated as random effect. Differences over time (a three-level factor) were tested using the Tukey Honest Significant Difference *post hoc* test performed with the multcomp R software package (Hothorn et al., 2008). *p*-values were viewed as highly significant when *p* < 0.01 and as significant when *p* < 0.05. Permutation t-test (1,000 replications) was used to assess differences among clinical characteristics between intervention and control groups at T0.

## 3 Results

The independent variables were Group assignment (Preterm Intervention and Preterm Control), and Time, measured at three different time points (T0, before the intervention, T1 after the intervention, and T2 at 3 months corrected age). The dependent variables were Global GMs (see Tables 2, 3) and Detailed GMs (see Tables 4, 5).

**Table 4:** GMs detailed scores (optimality scores, GMOS) at T0, before the intervention, and at T1 after the intervention.

Group	Time point	Mean GMOS	NA	Total
Intervention	T0	38.28	1	28
Intervention	T1	37.77	3	26
Control	T0	34.76	2	25
Control	T1	35.81	2	25

GMOS, general movements optimality scores; NA, not available.

**Table 5:** GMs detailed scores (motor optimality scores, MOS) at 3 months corrected age.

Group	Time point	Mean MOS	NA	Total
Intervention	3 months	26.24	4	25
Control	3 months	25.66	6	21

MOS, motor optimality scores; NA, not available.

Although a visual inspection suggests that the control group included relatively more infants with CS and PR, this difference was not statistically significant when assessed using appropriate statistical analyses.

Before the intervention, at baseline T0, there were no significant differences between the Control and Intervention Groups in terms of their main clinical characteristics, as assessed via a permutation *t*-test (1,000 replications). The *p*-values are reported in Table 1.

The following tables present the raw mean Global and Detailed scores at the different timepoints.

No significant differences between the two groups at T0, before the intervention, in terms of either the Global or Detailed GMs scores.

The non-linear mixed-effects model revealed that the intervention had a significant effect on GMs global scores, *p* < 0.05 (see Table 6), while accounting for time and the time × group interaction. Similarly, there was a marginally significant effect of the intervention on GMs detailed scores, *p* = 0.06 (see Table 6). In addition, time had a significant effect on the Global score between T0 (before the intervention) and T2 (at 3 months corrected age), *p* < 0.05 (see Table 6). However, no significant effect of time was found for GMs detailed scores, and the interaction term was not significant for either the Global or detailed scores (see Table 6).

**Table 6:** Linear mixed effect models for global and detailed scores.

Dependent variable	Estimate	Standard error	*t* value	Pr(>|t|)
GMs global scores				
Intervention	−0.23801	0.11926	−1.996	0.0496 *
Time	0.21582	0.08794	2.454	0.0194 *
Group*Time	0.04133	0.13248	0.312	0.7569
GMs detailed scores				
Intervention	−0.08853	0.04709	−1.880	0.0637^
Time	0.02889	0.03770	0.766	0.4479
Group*Time	0.05389	0.05576	0.966	0.3392

* Significant differences (*p* < 0.05); ^marginally significant differences (0.1 < *p* < 0.05).

Although we collected video recordings and the infants participated in the follow-up at T2, there were cases where the recordings were not suitable for evaluation. These missing evaluations were included with the data for preterm infants who did not have their last measurement at 3 months and are reported as NA in Tables 3, 5.

Supplementary Table S1 provides comprehensive information about the clinical characteristics of the Intervention and Control Groups, including the median, percentile, and range values. It also provides a more detailed account of the Cramped-Synchronized (CS), Poor Repertoire (PR), and Normal (N) GMs assessments.

## 4 Discussion

While promising evidence has been gathered demonstrating the short-term effects of early acoustic interventions, based on exposure to parents’ voices or live or recorded music in the NICU (Haslbeck et al., 2020; Kostilainen et al., 2021; Lordier et al., 2019), the longer-term impacts on preterm infant’s cognitive, linguistic, emotional, and general neurobehavioral development are still discordant (Filippa et al., 2024a; Haslbeck et al., 2021; Kostilainen et al., 2023; Lejeune et al., 2019; McGowan et al., 2024).

In the current study, we demonstrated a potential sustained effect of live maternal singing and speech on the neurobehavioral development of preterm infants, as measured via the General Movement Assessment, at term equivalent age and 3 months corrected age.

The current findings align with previous research. In a 2014 study (Picciolini et al., 2014) with 71 preterm infants, the recorded maternal voice was transmitted via bone conduction. At 3 months corrected age, the infants in the intervention group obtained significantly higher GMs scores (Ferrari et al., 1990) and higher scores on a neurofunctional assessment (Picciolini et al., 2022) than the control group. No significant differences were detected at 6 months corrected age.

However, although the outcomes appear to align with our own research, the undergoing mechanisms might be different.

In the present study, the intervention was a live, infant-directed vocal interaction, in which the mothers would have modulated their voices based on behavioral cues received from their preterm infants (Filippa et al., 2018), for example by amplifying the emotional content of their vocalizations in response to signs of positive communication on the part of their infants (Filippa et al., 2019a, 2019b, 2020). Moreover, mothers who actively engage in live infant-directed vocal interaction showed reduced levels of anxiety (Arnon et al., 2014), increased synchrony with their baby (Palazzi et al., 2020) and higher oxytocin levels (Filippa et al., 2023a; Filippa et al., 2023b; Hirschel et al., 2023), with potential knock-on effects on their sensitivity.

Given that previous research indicates positive associations between increased maternal sensitivity and the characteristics of infants’ spontaneous movements (Lev-Enacab et al., 2022), it is possible that the long-term beneficial effects of the present intervention rely on enhanced maternal sensitivity to the infant’s communicative cues. Interestingly, the quality of infants’ spontaneous movements at 3–4 months has been positively correlated with maternal sensitivity in terms of how mothers physically engage with their infants, but not in relation to the mother’s vocalizations (Lev-Enacab et al., 2015), which was the core focus of the present study. Future research is needed to disentangle the interactional mechanisms underlying the long-term effects of early contact between parents and their offspring.

Another potential mechanism linking the specific effects of the present intervention with general movements involves the brain responses of preterm infants to maternal speech and singing. Maternal singing activates key neural networks in newborns, including sensorimotor and salience regions, which play crucial roles in motor coordination, attention, and memory (Loukas et al., 2024). These regions are integral to the neural pathways underlying the production and refinement of general movements, suggesting that auditory stimulation from maternal singing directly supports the maturation of sensorimotor integration in preterm infants. Additionally, rhythmic components of singing are highly predictive and structured, providing an optimal stimulus for infant motor responses. During development, infants naturally respond to singing with rhythmic movements, indicating an innate link between auditory and motor systems (Zentner and Eerola, 2010). This synchronization highlights how musical stimuli, such as maternal singing, can engage motor pathways. Similar effects have been observed with maternal speech. Newborns show right-hemispheric brain responses to their mother’s voice, with activation patterns involving premotor and supplementary motor areas. These areas are essential for planning and coordinating movements, further supporting the connection between maternal vocalizations and motor development (Beauchemin et al., 2011).

Taken together, these findings highlight the potential brain mechanisms underpinning the effects of maternal singing and speech on the observed improvements in GMs in preterm infants.

On the other hand, our results are not consistent with those of earlier research in which music therapy in addition to kangaroo care versus kangaroo care alone had no significant impact on GMs scores (Span et al., 2021). This was possibly due to the post-natal age at which the preterms received the therapy, given that preterm infants with a post-natal age of <7 days obtained lower GM optimality scores than those with a post-natal age of >7 days. In the present study, the preterms were required to have attained stability before starting the intervention (for details see the Method section), and post-natal age at testing was very variable, ranging from 15 to 39 days in the intervention group and from 21 to 43 in the control group. Based on the above-mentioned findings of Span et al. with a larger sample, which prompted the authors to recommend postponing early live acoustic interventions until 7 days after birth, it is plausible to hypothesize that live vocal or musical intervention may lead to more favorable outcomes in stable preterm newborns, starting 1 or 2 weeks after birth. Nevertheless, further trials are needed to identify the optimum post-natal age for initiating parent-driven vocal and musical interventions.

Finally, the positive effects of the present vocal intervention inherently rely on parents’ intuitive ability to care for their offspring. In previous studies, we demonstrated that live maternal singing and speech contribute to stabilizing preterm infants. This stabilization, in turn, promotes better attending behaviors and improved organization of the newborn’s states (Filippa et al., 2013). Furthermore, live maternal interactions help to structure the behavioral organization of preterm infants, leading to an increase in their self-touching behaviors, such as hand-to-head movements (Filippa et al., 2020). Hand movements directed towards the head, face, and mouth are recognized as distinct self-regulation behaviors deployed by preterm newborns to modulate their levels of arousal (Als et al., 2005) and are viewed as crucial attempts at self-control or self-comforting. These basic interactive mechanisms lead to an increase in calm alert states accompanied by homeostatic behaviors, which impact in turn on maternal emotional availability (Filippa et al., 2019a) and on parents’ ability, for example, to modulate their voice in interactive contexts (Filippa et al., 2018; Saliba et al., 2020).

Furthermore, early intervention with the active involvement of parents, during a sensitive period for the development of the relationship between mothers and their preterm born infants, impacts on the preterm infants’ quality of movement and, consequently, on maternal responses and mutual regulation during early interactions (Lev-Enacab et al., 2022). Parents likely interpret regular, continuous, smooth, and fluent movements by their infants as an endearing signal of willingness to interact, which in turn may prompt more attuned maternal behavior (Kringelbach et al., 2016; Lev-Enacab et al., 2015).

Taken together, these observations point up specific basic mechanisms that also involve the modulation of the neuroendocrine system (Filippa et al., 2021b), the autonomous nervous system (Filippa et al., 2022b; Porges et al., 2019), and the brain (Filippa et al., 2023a; Welch et al., 2020b), thereby laying the ground for long-term and sustained effects on the development of newborns and children (Welch et al., 2020a).

The long-term, sustained effects of the early intervention tested in this study may be viewed as relatively certain, given that the intervention was largely based on the activation of biologically rooted and evolutionarily conserved parental behaviors and sensitivities (Rilling and Young, 2014), and given that it was implemented during a critical period in the brain development of preterm babies, during which the infant is particularly attentive and sensitive to acoustic stimuli (Benasich et al., 2014; Musacchia et al., 2017). Within a protective cycle of reciprocity, whereby parents both modulate and are affected by their infants’ behaviors, infant conspecifics can trigger protective parental responses, shielding offspring from the impacts of changes in transcriptional regulation within specific brain regions associated with parenting (Stolzenberg and Mayer, 2019).

### 4.1 Limitations

We are limited in our ability to generalize the results of this study due to the small sample size. A key risk of a limited population is that the impact of a few individual positive responses to the intervention can disproportionately influence the overall effects. Additionally, the potential value of including a simple measure of the time mothers spent with their babies in the NICU in future studies to better account for the overall maternal engagement. However, as noted in the statistical analysis section, the robustness of the RCT design and the types of analyses used help ensure the reliability of the results. Another limitation to the generalizability of these findings is the relatively stable health condition of the recruited preterm newborns. As specified, only infants without congenital anomalies and atypical brain malformations were included. Further research should include more fragile preterm infants, with a view to assessing how an environment enriched with maternal stimuli (Cioni et al., 2011) may modify atypical brain development trajectories.

An additional constraint of this study is the lack of assessment of the newborn infants’ overall language exposure and general interactional contact during and after hospitalization. Future studies should accurately assess, at various time points both during and after hospitalization, the average amount of adult words to which babies are exposed during the first months of life and the amount of time that dyads spend interacting. In the long term, the family’s musical habits should also be monitored (Politimou et al., 2018), given that the musical environment in the home in early infancy is known to have an impact on children’s language development (Franco et al., 2022).

Finally, our longitudinal analysis could have usefully been supplemented by additional forms of assessment. Future research should include neuroimaging techniques, which could provide insights into the underlying physiological mechanisms that contribute to GMs-related development, as well as assessment of mother–infant attunement as an outcome measure of efficacy for these forms of early intervention. Integrating multiple assessment approaches will further enhance our understanding of the evolution of GMs and provide a more complete picture of developmental pathways.

## 5 Conclusion

In the current study, we observed potential sustained effects of live maternal singing and speech on the neurobehavioral development of preterm infants, as measured by the General Movement Assessment, at term equivalent age and 3 months corrected age.

Supporting and enhancing parental speech and singing directed toward preterm infants has the potential not only to foster their linguistic development (McGowan et al., 2024) but also to stimulate sensorimotor integration, positively impacting neurobehavioral development as assessed by general movements. There is strong rationale for promoting early contact and interaction through infant-directed singing and speech within the framework of infant-and family-centered care (Filippa and Kuhn, 2024). Ensuring that caregivers and healthcare professionals are equipped to understand and implement early vocal and interactive strategies can improve outcomes for both infants and their families.

Future research should carefully control for interactional periods in the dyads during and after hospitalization and evaluate the effects of early parental intervention on parents’ brain structures and behaviors, at multiple levels (neuroendocrine, neural, and behavioral), over the first weeks and months of life, while parents are interacting with and caring for their preterm infants. Changes in parental behaviors, combined with specific modifications to infant brain functionality, thanks to the early intervention, may enhance the long-term developmental trajectories of this at-risk population.

## Data Availability

The original contributions presented in the study are included in the article/supplementary material, further inquiries can be directed to the corresponding author.
