# Supplementary material for: Maternal singing and speech have beneficial effects on preterm infant’s general movements at term equivalent age and at 3 months: an RCT
Source: Front Psychol. 2025 Jan 29;16:1536646. doi: 10.3389/fpsyg.2025.1536646 (PMC11815593; doi:10.3389/fpsyg.2025.1536646)
Supplement: Supplementary file 1 [file Data_Sheet_1.docx]

**Supplementary Material**

S1. Detailed description of General Movements (GMs) Assessment

General Movements are spontaneous, non-goal-directed movements that involve the entire body. They serve as an essential indicator of the integrity of the developing nervous system and are characterized by their variability, complexity, and fluidity. When the nervous system is impaired, General Movements lose their complex and variable character and exhibit a poor repertoire or become cramped-synchronized or chaotic. Fidgety movements can be either abnormal or absent.

**Cramped-Synchronized GMs:** This abnormal pattern is observed from preterm age onward. Movements appear rigid and lack the normal smooth and fluent character; all limbs and trunk muscles contract and relax almost simultaneously (Ferrari et al., 1990). If this abnormal pattern is observed consistently over a number of weeks, it is of high predictive value for the later development of spastic cerebral palsy (Ferrari et al., 1990).

**Poor Repertoire GMs:** This abnormal GM pattern occurs during preterm, term, and early post-term age. The sequence of successive movement components is monotonous, and movements of different body parts do not occur in the complex way seen in normal GMs (Ferrari et al., 1990; Einspieler et al., 1997). Poor repertoire GMs can be followed by normal, abnormal, or absent fidgety movements, hence their predictive value is rather low.

**Abnormal Fidgety Movements:** These movements resemble normal fidgety movements, but their amplitude, speed, and jerkiness are moderately or greatly exaggerated.

**Absence of Fidgety Movements:** If fidgety movements are never observed from 9 to 20 weeks post-term, we call this abnormality "absence of fidgety movements," which is highly predictive for later neurological impairment, particularly for cerebral palsy, both the spastic (Prechtl et al., 1997) and dyskinetic forms (Einspieler et al., 2002).

Table S1. Clinical Characteristics of the Intervention and Control Groups (median, P25-75 and range)

| Gestational Age at Birth (weeks), *Median (25/75)* | 29 (28/31) | 30 (28/31) |
| --- | --- | --- |
| Birthweight (grams)*, Median (25/75)* | 1243 (1022/1291) | 1330 (1087.5/1495) |
| Apgar Score at 5 Min*, Median (25/75)* | 5 (3/6) | 6 (5/7) |
| Mother’s Age (years)*, Median (25/75)* | 36 (35/40) | 35.5 (31/39) |
| Gestational age at Intervention Start (weeks)*, Median (25/75)* | 33 (33/35) | 34 (33/35) |
| Weight at Intervention Start (weeks)*, Median (25/75)* | 1600 (1410/1990) | 1796 (1491/1999) |
| Post Natal Age at Intervention Start*, Median (25/75)* | 24 (15/39) | 29 (21/43) |

Table S2. The proportion of Cramped-Synchronized (CS), Poor Repertoire (PR) or Normal (N) GMs at the three timepoints, at T0, before the intervention, at T1, term equivalent age, and at T2, at 3 months

|  | T0 | T1 | T2 |
| --- | --- | --- | --- |
| Intervention (n = 29) | Ntot = 28  CS=0; PR=6; N=22; | Ntot= 25  CS=0; PR= 8; N=17; | Ntot= 24  CS=0; PR=0; N=24; |
| Control (n = 27) | Ntot= 25  CS=1; PR=7; N=17; | Ntot= 24  CS=1; PR=7; N=16; | Ntot= 19  CS=0; PR=2; N=17; |
| Total (56) | Ntot= 53  CS=1; PR=13; N=39; | Ntot= 49  CS=1; PR=15; N=33; | Ntot=43  CS=0; PR=2; N=41; |
